# Statistical Complexity Analysis of Sleep Stages

**DOI:** 10.3390/e27010076

**Published:** 2025-01-16

**Authors:** Cristina D. Duarte, Marianela Pacheco, Francisco R. Iaconis, Osvaldo A. Rosso, Gustavo Gasaneo, Claudio A. Delrieux

**Affiliations:** 1 Departamento de Física, Instituto de Física del Sur, Universidad Nacional del Sur-Consejo Nacional de Investigaciones Científicas y Técnicas (CONICET), Bahía Blanca 8000, Argentina; mpacheco@icic-conicet.gob.ar (M.P.); francisco.iaconis@uns.edu.ar (F.R.I.); ggasaneo@uns.edu.ar (G.G.); 2 Departamento de Ingeniería Eléctrica y Computadoras, Instituto de Ciencias e Ingeniería de la Computación, Universidad Nacional del Sur-Consejo Nacional de Investigaciones Científicas y Técnicas (CONICET), Bahía Blanca 8000, Argentina; cad@uns.edu.ar; 3 Instituto de Física, Universidade Federal de Alagoas UFAL, Maceió 57072-900, Brazil; oarosso@conicet.gov.ar

**Keywords:** permutation entropy, statistical complexity, generalized weighted permutation entropy, sleep stages

## Abstract

Studying sleep stages is crucial for understanding sleep architecture, which can help identify various health conditions, including insomnia, sleep apnea, and neurodegenerative diseases, allowing for better diagnosis and treatment interventions. In this paper, we explore the effectiveness of generalized weighted permutation entropy (GWPE) in distinguishing between different sleep stages from EEG signals. Using classification algorithms, we evaluate feature sets derived from both standard permutation entropy (PE) and GWPE to determine which set performs better in classifying sleep stages, demonstrating that GWPE significantly enhances sleep stage differentiation, particularly in identifying the transition between N1 and REM sleep. The results highlight the potential of GWPE as a valuable tool for understanding sleep neurophysiology and improving the diagnosis of sleep disorders.

## 1. Sleep Stage Interpretation and Analysis

Sleep and waking states are periodic biobehavioral features that are characterized by changes in brain electrical activity, manifested as alterations in consciousness, reduced sensory responsiveness, decreased muscle tone, and relative inactivity [1]. This process has an important adaptive function in the homeostasis of mammals, guaranteeing their survival [2]. In humans, alterations in the quality, quantity, and patterns of sleep (i.e., sleep disorders) negatively impact the individual’s overall health and functionality [1,3,4,5]. Sleep disorders are extremely common in the general population [1,5].

Normal human sleep consists of two primary states, rapid eye movement (REM) and non-REM, each with distinct stages and characteristics. Furthermore, non-REM sleep includes several stages, ranging from light to deep sleep, while REM sleep is associated with vivid dreaming and brain activity patterns similar to those of wakefulness. These sleep stages undergo age-related changes and are influenced by factors such as sleep–wake history, circadian timing, and pharmacological agents, all of which impact the distribution and quality of REM and non-REM sleep [6]. Accurately assessing these sleep stages is essential for understanding various health conditions, including insomnia, sleep apnea, and neurodegenerative diseases such as Parkinson’s and Alzheimer’s [7].

For example, disruptions in deep sleep have been linked to cognitive decline [8,9], while abnormalities in REM sleep may indicate certain mood disorders [10,11] or post-traumatic stress disorder [12,13]. This analysis requires robust methods to discriminate sleep stages from physiological signals. The electrical signals of the brain recorded by EEG are the most used technique to understand the microstructure of sleep waves [14]. By analyzing these signals, we can identify the different stages of sleep—from light sleep (N1, N2) to deep sleep (N3) and REM stages—and assess their duration and how a person transitions between them. This allows for the accurate identification of sleep disorders, which can lead to more effective diagnostic and treatment interventions [15].

The EEG signal reflects the electrical activity of the brain, which forms a chaotic, non-linear temporal signal whose features has been studied from the standpoint of statistical complexity, with efforts aimed at developing automatic and precise methods for classifying sleep stages. Among the earliest studies to apply statistical complexity analysis in sleep stages is that presented in [16]. This paper discusses how permutation entropy (PE) can be applied to analyze EEG sleep signals in artifact-free segments of 30 s, providing measures that help identifying different sleep stages and detecting anomalies, such as those related to neurological conditions. The study shows that PE is useful for characterizing sleep EEG and offers a computationally efficient tool to measure the complexity of brain activity during different sleep stages, contributing to a better understanding of sleep physiology and potential disorders. In [14], the authors explore the statistical complexity of EEG signals during sleep, particularly focusing on the Cyclic Alternating Pattern (CAP), which is a key feature of the sleep microstructure. The study applies complexity-based features like fractal dimension and sample entropy to evaluate these activations and compares them to non-activation periods in NREM sleep. Their findings suggest that different subtypes of CAP activations (A1, A2, and A3) have distinct levels of EEG complexity, with A3 showing the highest complexity across all sleep stages. The ability of ordinal patterns to detect minute features in EEG signals is exposed in [17]. A modified PE assessment is here understood as a measure of distance from white noise, with the support of a specific statistical hypotheses test in which critical significance values are provided. That paper presents the first evidence of automated analysis that goes beyond expert human assessment since the method is able to detect delta wave patterns and graphic features which are only visible on a time scale of several seconds. At the same time, the “distance from white noise” measure serves as an indicator of sleep depth, in which the awake state is treated as the nearest to white noise.

Building on these advancements, the statistical complexity derived from Jensen–Shannon divergence has emerged as a robust alternative for capturing the intricate interplay between randomness and order in EEG signals. Unlike measures such as PE or sample entropy that primarily quantify disorder, this statistical complexity excels in identifying intermediate levels of organization within the signal. This sensitivity allows it to distinguish not only between wakefulness and sleep but also among different sleep stages with greater precision [18]. In comparison, approximate entropy (ApEn) is effective in measuring signal regularity and has been used for detecting anomalies such as epileptic seizures. However, it is sensitive to parameter selection and suffers from issues such as a bias toward shorter data lengths, which can lead to inconsistent results across datasets [19].

Lempel–Ziv complexity (LZC), on the other hand, offers a straightforward algorithm that assesses the randomness of finite sequences by the number of steps required to generate them, making it particularly suitable for real-time applications like depth-of-anesthesia monitoring [20]. However, LZC often fails to account for intermediate levels of complexity, as it primarily evaluates disorder without distinguishing structured chaotic dynamics from noise [21]. The ability of statistical complexity to integrate measures of entropy and disequilibrium positions it as a more comprehensive tool. Unlike ApEn and LZC, statistical complexity not only distinguishes structured chaotic dynamics from random patterns but also quantifies the nuanced transitions between these extremes, providing deeper insights into the non-linear dynamics of EEG signals [18].

The use of machine learning methods in classifying sleep stages is also proposed in [22], specifically to score sleep stages using single-channel electrooculogram (EOG) signals. These signals are analyzed using several features (discrete wavelet transform, spectral entropy, moment-based measures, composite multiscale dispersion entropy, and autoregressive coefficients). The discriminative power of these features is assessed through ANOVA tests, and feature reduction is applied to decrease model complexity and retain the most discriminative features. Finally, sleep stage classification is performed using Random Under-Sampling Boosting (RUSBoost), random forest (RF), and support vector machines (SVMs). On a similar setting, in [23], the authors combine entropy features (including fuzzy measure entropy, fuzzy entropy, and sample entropy), with a multi-class SVM to classify sleep stages. The method analyzes 30 s segments of Fpz-Cz and Pz-Oz EEG signals, together with horizontal EOG signals from the Sleep Telemetry Study dataset [24].

Given the widespread success of ordinal patterns and statistical complexity-based signal analysis, there has been a recent influx of alternative techniques that may outperform the more general methods. In particular, in [25], the authors propose ensemble improved PE and multiscale ensemble improved PE for taking into account both permutation relations and amplitude information. The proposed methods show better discriminating power and robustness, specifically in the analysis of EEG signals. In our study, we pursue a comparable objective: to analyze and classify sleep stages from polysomnographic data by training a classifier using novel statistical complexity measures as features, specifically generalized weighted permutation entropy (GWPE).

While deep learning methods excel at automating feature extraction, their black-box nature and high resource demands can make them less suitable for EEG analysis, where interpretability and performance with minimal data are often priorities. Our results outperform traditional analysis based on ordinal patterns, achieving accuracies comparable to those obtained with deep learning [26]. The advantage of using feature-engineered white-box models in this context (as opposed to data-driven deep learning models) is that handcrafted features are more interpretable in neurophysiological terms, providing clearer insights into the underlying neural mechanisms, which is crucial in fields like neuroscience and clinical diagnosis in which model explainability is required. Additionally, feature-engineered models are usually less computationally intensive, can work effectively with smaller datasets, and are much easier to re-train, making them a more practical choice in scenarios where labeled EEG data are varying or limited.

## 2. Materials and Methods

### 2.1. Database Description

The dataset used is obtained from Bob Kemp’s publicly available database on Physionet, linked to the Sleep Telemetry Study [24] already mentioned, which consists of a set of polysomnographic signal recordings. That study investigated the effects of temazepam on sleep in 22 Caucasian men and women, ages 18 to 79, 7 men (mean age 35.71) and 15 women (mean age 42.26). The subjects had mild difficulties falling asleep, were not taking any other medications, and were healthy subjects. This dataset is made up of a total of 44 recordings in the hospital, corresponding to two nights, that is, two recordings per subject. The polysomnographic recordings lasted nine hours, including the location of the Fpz-Cz and Pz-Oz electrodes, and were performed after temazepam ingestion or after placebo ingestion. For the present work, the corresponding records are included for subjects between 18 and 40 years old after taking the placebo. The dataset is made up of 12 polysomnographic records of 12 subjects (total mean age for men and women 25.83, standard deviation: 6.55, sample rate 100 hz). More specifically, the study is composed of 8 women (mean age women 27.87, standard deviation: 7.20) and 4 men (mean age men 21.75, standard deviation: 1.71). We use only the Fpz-Cz channel of the EEG signal. These recordings include expert annotations of sleep stages, labeled in 30-s intervals, which the experts term *epochs*, each consisting of 3000 data points.

### 2.2. Generalized Probabilities of Ordinal Patterns

Permutation entropy (PE) [27] is a robust, non-linear time series analysis method that quantifies the complexity and randomness of a system, having gained widespread adoption due to its simplicity, computational efficiency, and ability to capture dynamic patterns in diverse fields such as neuroscience, finance, and engineering. The speed and robustness that characterize PE make it particularly useful for handling large datasets without requiring extensive preprocessing or parameter tuning [17].

The computation of PE involves transforming the time series into a sequence of symbols known as ordinal patterns (OPs). These OPs are characterized by two key parameters: the length of the pattern, *D*, and the time delay τ, which denotes the interval between the samples in the signal used to define the patterns. Given a time series $x_{t}$ where t=1,⋯,T, overlapping segments *s* of length *D* are taken with a τ interval between consecutive samples. This results in a total of T−(D−1) segments. Within each segment, the *D* samples are labeled with ordinal numbers 1,2,⋯,D according to their ordinal relationships with other values in the same segment. Then, each segment is labeled with an OP, which is one permutation π among the D! possible permutations of *D* ordinal values. In a given signal, the relative frequency (or empirical probability) of each OP $\pi_{i}$ is then

(1) $$p\left(\pi_{i}\right)=\frac{\sum_{s\;,\;\pi_{s}=\pi_{i}}1}{\sum_{s}1},$$

where *s* represents each of the sequences, and $\pi_{s}$ is the corresponding OP of *s*. The probability distribution function (PDF) of the D! OPs is then $P_{\pi }=\left\{p\left(\pi_{1}\right),\ldots ,p\left(\pi_{D!}\right)\right\}$. From this PDF, the PE of the signal can be computed using Shannon entropy:

(2) $$S=-\sum_{i}^{D!}p\left(\pi_{i}\right)log(p\left(\pi_{i}\right).$$

It can be shown that *S* takes its minimum value of 0 when there is total certainty about the series, i.e., a given permutation has probability one. The maximum value corresponds to the case where uncertainty is maximized (log(D!)), which corresponds to a uniform probability distribution $P_{U}=\left\{p_{i}=1/M,i=1,\ldots ,M\right\}$, where all states have the same occurrence probability.

As mentioned above, alternative complexity measures, such as multiscale entropy or fractal dimension, lack the capability to jointly account for randomness and chaotic patterns, which is crucial in nonstationary signals like EEG that exhibit both stochastic and deterministic behaviors. Statistical complexity addresses this gap by integrating entropy with disequilibrium, defined as the product of normalized Shannon entropy *H* and disequilibrium *Q*:

(3) $$C=Q\left[P_{\pi },P_{u}\right]H\left[P_{\pi }\right],$$

where normalized Shannon entropy *H* is defined as $S/S_{max}$, disequilibrium *Q* is defined in terms of the Jensen–Shannon divergence $Q=Q_{0}\cdot D\left[P_{\pi },P_{u}\right]$, and the divergence *D* provides a measure of the similarity between two probability densities. Here, the PDF is compared with the uniform distribution:

(4) $$D\left[P_{\pi },P_{u}\right]=S\left(\frac{P_{\pi }+P_{u}}{2}\right)-\left(\frac{S\left[P_{\pi }\right]+S\left[P_{u}\right]}{2}\right)$$

The lowest complexity value occurs when the system is either entirely random or entirely ordered. When the system is in a state of maximal disorder, there is no structural information, and the complexity is low. When the system is in a highly ordered state, it is fully predictable, meaning there is no uncertainty in the pattern distribution, which also results in low complexity. Entropy and complexity can be articulated together in the complexity–entropy causality plane. In [28], it is shown how to simultaneously quantify both the information content and structural complexity of a time series, distinguishing between stochastic noise and deterministic chaotic behavior, leading to many applications in analyzing and characterizing time series and signals from physiology, physics, and many other fields.

While PE has been effective in various applications, the resulting assessments may have limitations when the local amplitude of the time series is very uneven since smaller fluctuations should exert less influence in the overall analysis. To this avail, in [29], the authors propose weighted PE (WPE), which assigns more significance to OPs with higher amplitude fluctuations. This is accomplished by incorporating the local variance of each OP as a weighing factor of its contribution to the overall empirical probabilities. This modification allows WPE to capture both the ordinal structure and amplitude information of a time series, making it more suitable for applications where amplitude plays a key role. Furthermore, in [30], the authors propose the generalized weighted PE (GWPE), which also considers an entropic index *q* that allows to discern between the effects of small or large fluctuations in the overall complexity of the signal. The associated probability distribution is then

(5) $$p\left(\pi_{i},q\right)=\frac{\sum_{s,\pi_{s}=\pi_{i}}w^{q/2}}{\sum_{s}w_{s}^{q/2}}.$$

GWPE coincides with WPE for q=2, and with the original PE for q=0, making the notation and parameters compatible with other nonextensive measures (e.g., Tsallis entropy) or nonstationary fluctuation analysis (e.g., multifractal spectrum). As with other nonextensive/nonstationary models, entropic indices are used to generalize the concept of entropy to systems with long-range interactions or memory effects, fractal structures, or complex correlations, providing a more accurate description of their statistical behavior and properties. In our particular context, the relevance of the use of GWPE lays in how different ordinal patterns are weighted, given that negative *q* values enhance the contribution of small fluctuations in the OP distribution, while positive values enhance the contribution of large fluctuations.

In this study, we are focused on determining how entropic indices may enhance the ability of a PE-based polysomnographic signal analysis. As already mentioned, this approach enables a more flexible and detailed evaluation of the disorder and structure of the time series. A likely interpretation of Equation (5) is that now PE is a function of the entropic index and thus signals can be assessed according to this function. In particular, instead of evaluating this PE function for signals, or characterizing them in the complexity–entropy causality plane as in [30], our idea is to use the generalized probabilities $p(\pi_{i},q)$ of the OPs as features for classification, and study the ability of the resulting classifier, as compared to the standard PE, to distinguish the different sleep stages as described in the next subsection.

### 2.3. Data Analysis

For each subject, the EEG signal is segmented according to the labeled epochs, with each epoch corresponding to a specific sleep stage. The numbers of epochs across all subjects are N1 = 885, N2 = 5281, N3 = 1995, R = 2599, and W = 803, for a total of 11,563 epochs. Given the uneven distribution of epochs, we downsample the N2 class by randomly dropping half of the epochs, and we perform a data augmentation procedure with N1 and W (waking state), creating new epochs in the middle of each pair of consecutive epochs belonging to the same class (i.e., starting at the midpoint of the first epoch and ending at the second epoch). Furthermore, for each sleep cycle, we remove the two boundary epochs marking the transition from one sleep phase to the next. The final number of epochs in each state is N1 = 978, N2 = 2247, N3 = 1643, R = 2444, and W = 1017. The total number of epochs across all states is 8329.

In calculating the generalized probabilities $p(\pi_{i},q)$ for each subject and epoch, we use a time delay of τ = 1, and a segment length D = 4. These parameter values are chosen based on [18], which demonstrate their effectiveness in characterizing sleep stages. The index *q* is varied in integer values in the [−10,10] interval, for a total of 21 *q* values. Using these values, the generalized probabilities of each of the D! ordinal patterns in each epoch are computed, giving, in total, 504 features (24×21) for each epoch. Thus, the shape of the dataset for training the classifier is 8329×505, the former being the rows corresponding to each epoch, and the latter the columns, including the 504 features plus the corresponding sleep stage label.

With this dataset, several classification models are trained, including random forest, support vector machines, and XGBoost. In each case, the dataset is split into 85% for training and 15% for testing. The training data are further divided using 5-fold cross validation, hyperparameter tuning procedures are performed, as well as feature selection for evaluating the effectiveness of the different features in distinguishing sleep stages W, N1, N2, N3, and REM. The optimal hyperparameters for each model are determined using RandomizedSearch implemented through the scikit-learn library [31], with 75 random permutations of parameter values. The performance of the best hyperparameters is evaluated based on accuracy. The above procedures are applied to the full set of features (corresponding to the generalized probabilities) and with only the 24 features corresponding to standard PE (q=0). In addition, the PE and complexity of the 21 *q* values are assessed.

## 3. Results

### 3.1. Classifier and Feature Set Comparison for Sleep Stage Classification

To illustrate the advantages of the generalized probabilities of the OPs in improving sleep stage classification, we train three algorithms to automatically classify the five stages. Here, we present the results only for XGBoost, which demonstrates better performance as compared to other widely used machine learning models like random random forests and support vector machines. The results of all the methods are presented in Appendix A.1 and Appendix A.2 for comparison. We evaluate the classification performance with different feature sets to identify which features best separate the sleep stages. Each algorithm is computed in the following order:

- Permutation entropy (PE) and complexity (C);
- Generalized weighted permutation entropy (GWPE) and complexity (GWPEC);
- The probability distribution function of the ordinal patterns (PDF);
- The generalized weighted probability distribution of the ordinal patterns (GWPDF).

**Figure 1:**
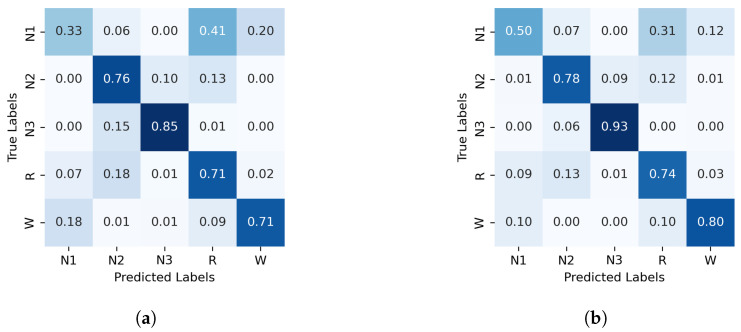
Normalized confusion matrix (%) for XGBoost algorithm classifier, with PE and C on the left and GWPE and GWPEC on the right as features. (**a**) XGBoost algorithm; PE and C as features, (**b**) XGBoost algorithm; GWPE and GWPEC as features.

**Figure 2:**
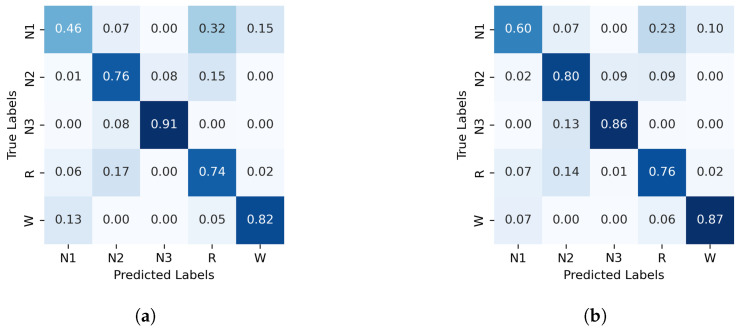
Normalized confusion matrix (%) for XGBoost algorithm classifier, with PDF on the left and GWPDF on the right as features. (**a**) XGBoost algorithm; PDF as features, (**b**) XGBoost algorithm; GWPDF as features.

The normalized confusion matrices for the classification of sleep stages using XGBoost, trained with PE and C, as well as GWPE and GWC, are presented in Figure 1. Similarly, Figure 2 shows the confusion matrices for the classification using the probability distribution of the ordinal patterns (PDF) and generalized weighted probability distribution (GWPDF). All confusion matrices are calculated using the classifier optimized through cross-validation. Furthermore, the matrices are generated after performing feature importance analysis on both the generalized weighted permutation entropy and complexity, as well as on the generalized weighted probability distribution of ordinal patterns. Confusion matrices for the other classifiers are available in Appendix A.1.

Table 1 summarizes the mean accuracy and standard deviation obtained from cross validation for XGBoost. Detailed performance metrics for random forest and support vector machine can be found in Appendix A.2. Feature importance analysis is conducted to identify the most relevant features that contribute to the model’s predictive performance. The results using GWPDF as features, as well as the results for GWPE and GWPEC as features, are presented in Table 2.

The importance of each feature is calculated based on the total accuracy gain of the feature when it is used in training. In Table 2 are presented, for both features sets, the features, the corresponding index *q* used in the calculation, and their relative importance. Only the top 10 features are displayed, ordered based on their impact on the classification accuracy. Using OP, the top 10 features contribute to 35% of the cumulative importance, whereas for GWPE, these same top 10 features contribute to 64% of the cumulative importance.

### 3.2. Behavior of PE, C vs. *q* and POs vs. *q*

**Figure 3:**
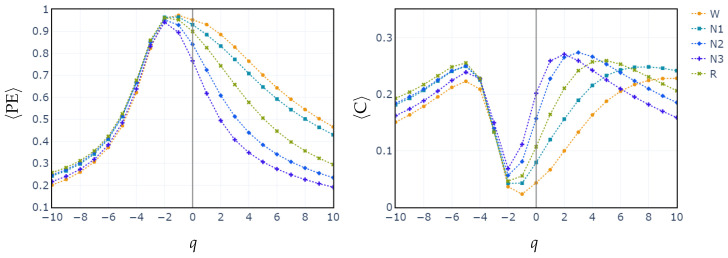
Median of values of generalized permutation entropy (**left**) and complexity (**right**) for *q* across all sleep stages.

To illustrate how the entropic index *q* exerts influence in differentiating between sleep stages, we compute the median values of PE and C for all subjects across each sleep stage, as a function of *q* parameter. The exploration of the *q* parameter is shown in Figure 3. This sheds light on how different values of *q* affect the separation of sleep stages based on generalized permutation entropy and complexity. In both cases, PE and C, for q<−1, the medians across all stages remain closely clustered. Starting from q=−1, the median values begin to diverge. This behavior is consistent with the feature selection performed in the cases of RF and XGBoost classifiers. In these cases, it is shown that entropy and complexity with positive exponents have the highest relative importance.

Additionally, we plot the median values of the probability distributions for the patterns [0123] and [3210]. We choose these patterns because they exhibit the highest relative importance in the XGBoost classification. We observe once again that the separation between the stages begins at q≥−1.

## 4. Discussion

The results show improvements in median accuracy when moving from standard PE and C to GWPE and GWPEC across all classifiers (see Table A1 in the Appendix A.2). The use of PDF as a feature set improves the classification as compared with the standard PE but does not fully capture the signal’s complexity.

GWPE allows for more accurate discrimination between different sleep phases, including the subtle transitions between stages such as N1 and REM. This enhanced capability is due to the fact that GWPE not only considers the order of patterns in the EEG signal as standard PE does but also weights these patterns according to their magnitude, allowing us to highlight the contributions of both small and large fluctuations. Specifically, negative values of *q* in GWPE increase the contribution of small fluctuations, while positive values increase the contribution of large fluctuations. This weighted approach provides a more detailed characterization of brain dynamics. In this context, this measure reflects the dynamic organization of neural activity during sleep.

As the individual progresses into deeper levels of sleep, cortical brain waves become increasingly synchronized. This synchronization increases the signal amplitude while reducing frequency, which significantly alters the probabilities of the ordinal patterns, particularly increasing the likelihood of monotonic patterns [0123] and [3210], common in deterministic and periodic signals. This feature explains the higher accuracies achieved for the N3 stage (as reflected in the confusion matrices across all feature sets) since this stage mainly contains low-frequency, high-amplitude delta waves, in which these monotonic patterns prevail.

**Figure A1:**
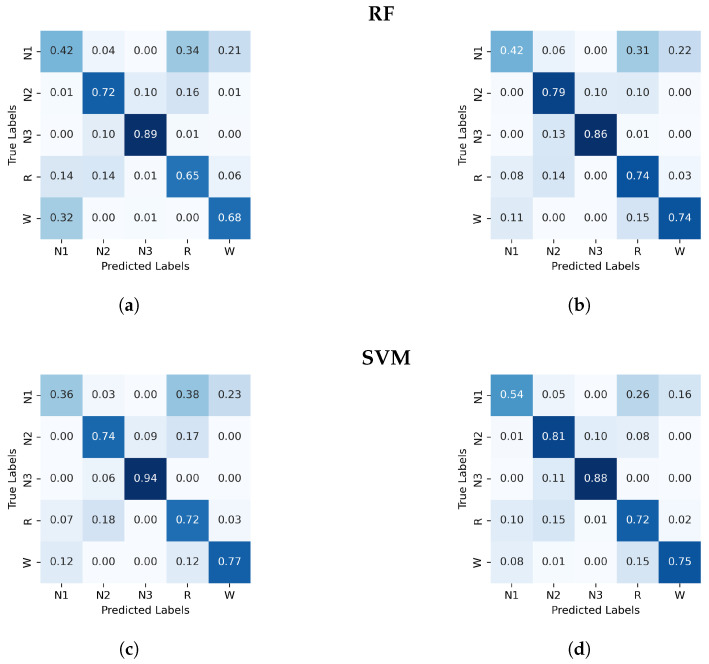
Normalized confusion matrix (%) for RF and SVM algorithms classifier, with PE and C on the left and, GWPE and GWPEC on the right as features. (**a**) RF algorithm; PE and C as features, (**b**) RF algorithm; GWPE and GWPEC as features, (**c**) SVM algorithm; PE and C as features, (**d**) SVM algorithm; GWPE and GWPEC as features.

**Figure A2:**
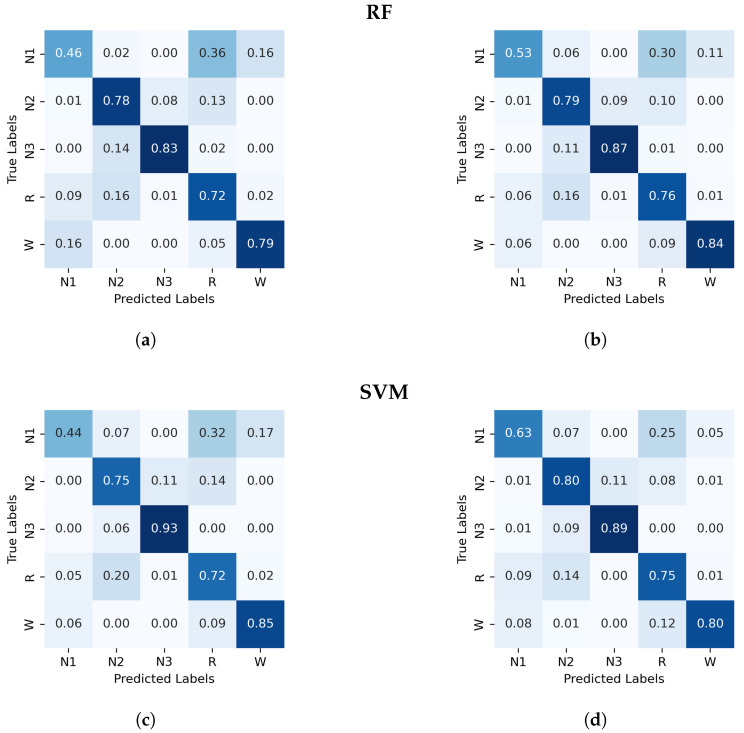
Normalized confusion matrix (%) for the RF and SVM algorithms classifier, with PDF on the left and GWPDF on the right as features. (**a**) RF algorithm; PDF as features, (**b**) RF algorithm; GWPDF as features, (**c**) SVM algorithm; PDF as features, (**d**) SVM algorithm; GWPDF as features.

For N2, the classification accuracy is generally high across the three algorithms using PE and C as features. However, adding the entropic index *q* further enhances the performance in both GWPE and GWPDF feature sets (see Figure 1, Figure 2, Figure A1 and Figure A2). Stage N2 is characterized by distinctive K-complexes (brief, large-amplitude spikes) and sleep spindles (12–16 Hz oscillatory waves). Considering signal amplitude likely improves the classifiers’ ability to detect these patterns by highlighting specific amplitude and frequency occurrences, thereby reducing the risk of misinterpretation as noise, which may occur when using only PE and C.

**Figure 4:**
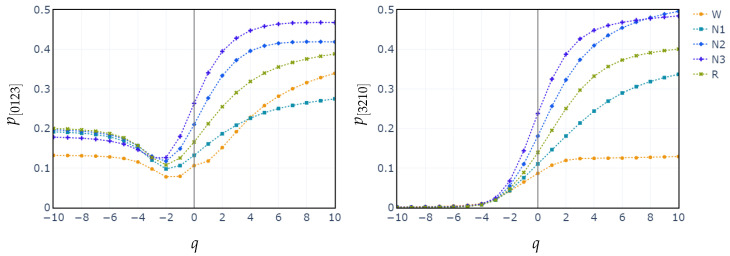
Median probabilities of the monotonic patterns [0123] and [3210] across all sleep stages for varying entropic index *q*.

In contrast, classifying N1 sleep remains challenging. During wakefulness, the EEG generally shows lower complexity due to regular alpha or beta waves. As the signal transitions into stage N1, there is an intermediate increase in complexity as alpha waves decrease and theta waves emerge. Thus, using PE as features, particularly distinguishing N1 from wakefulness and REM, obtains low classification accuracy due to overlapping EEG signal features. However, the use of generalized probabilities of ordinal patterns, weighted by the entropic index *q*, significantly enhances the classifier’s ability to distinguish N1 from other stages, leading to GWPE values that are distinct from W and REM. In REM sleep, the EEG signal is highly complex and disordered, with a mixture of theta and beta waves and increased signal variability, resulting in higher GWPE values (see Figure 3). The confusion matrices demonstrate a significant enhancement in N1 classification accuracy, increasing from 33% (Figure 1a) to 60% (Figure 2b) with the use of generalized probabilities. This improvement can be observed in two ways. First, as shown in Figure 3, N1 becomes more distinguishable from W and R at q≥−1 in PE and C. Second, as shown in Figure 4, the probability of weighted patterns captures finer distinctions between N1 and the other sleep stages. This suggests that incorporating both the variance of the EEG signal and the occurrence probabilities of each pattern may allow for deeper capture of the microstructure within sleep stages. Specifically, at values of q≥−1, there is a marked improvement in stage discrimination, suggesting that large fluctuations in EEG signals play a critical role in enhancing classification accuracy. This can be seen in Figure 3 and Figure 4, where distinctions among all stages are apparent.

The fact that XGBoost achieves the best performance can be attributed to an uneven distribution of the OP relative frequencies of each sleep stage in the feature space, which makes a boosting strategy more effective than the SVM geometric method or the RF bagging strategy. Moreover, an analysis of feature informativeness reveals a contrasting distribution in predictive power across the two feature sets. In the plain PE feature set, the ten most informative features capture only 35% of the total predictive performance gain of the classifiers, indicating a relatively dispersed distribution of informativeness across features. In contrast, in the GWPE feature set, the top ten features account for 65% of the total gain, suggesting a more concentrated distribution, where fewer features contribute a much larger amount of information relevant to the classification accuracy. As shown in Table 2, the entropic indices that achieve the highest relative importance in classifying sleep stages are those greater than or equal to −1. This applies to both the probability of ordinal patterns and the complexity and entropy measures (PE and C) derived from these probabilities. This observation points to differences in feature redundancy and offers insights into the complexity of feature selection and model optimization.

## 5. Conclusions

In this study, three classification algorithms (RF, SVM, and XGBoost) were evaluated to assess their performance with different feature sets, specifically examining the improvement in classification accuracy using GWPE. Our results indicate that these measures provide a more comprehensive understanding of the complexity of EEG signals compared to traditional permutation entropy and complexity. The use of the entropic index *q* was shown to be crucial for more effectively capturing the complexity of EEG signals, as it allows for more precise modulation of ordinal probabilities based on EEG fluctuations. This approach provides a more refined characterization of the dynamic patterns of brain activity. It is especially useful for distinguishing between sleep stages, such as N1 and REM, where brain activity is often similar. The use of generalized ordinal probabilities weighted by *q* contributed to improving the distinction between these phases, especially in values of q≥−1, suggesting that wide fluctuations in the EEG signal are a key aspect in differentiating them.

The ability to adjust ordinal probabilities to capture the non-linear dynamics and complexity of the EEG has potential applications both in the clinical diagnosis of sleep disorders and in the study of other complex signals and time series. In future work, it would be necessary to explore in depth the effects of different values of the entropic index *q* in other populations and conditions such as in subjects with specific sleep disorders. On the other hand, it would be valuable to investigate whether the integration of other physiological signals, in addition to EEG, could further complement and improve the classification results, creating more robust and accurate diagnostic tools in the field of sleep disorders.

## Figures and Tables

**Table 1:** Mean accuracy (Acc.) and standard deviation (SD) for the XGBoost classifier.

		Acc	SD
Features	PE and C	0.71	0.01
	GWPE and GWPEC	0.77	0.01
	PDF	0.74	0.01
	GWPDF	0.80	0.01

**Table 2:** Top ten features ranked by relative importance for XGBoost classifiers. The classifiers are trained with GWPDF (left) and GWPE and GWPEC (right). For both sets, the first column lists the respective feature, the second column presents the entropic index *q* used to calculate the feature, and the third column shows the relative importance of each feature.

OPs			GWPE and GWPEC		
Features	*q*	Relative Importance	Features	*q*	Relative Importance
$p_{\left[3210\right]}$	0	0.0868	*H*	1	0.1655
$p_{\left[0123\right]}$	1	0.0716	*H*	0	0.1028
$p_{\left[0123\right]}$	− 1	0.0355	*C*	3	0.0874
$p_{\left[1032\right]}$	2	0.0290	*C*	1	0.4366
$p_{\left[1032\right]}$	3	0.0286	*H*	−1	0.0595
$p_{\left[3210\right]}$	1	0.0285	*C*	0	0.0416
$p_{\left[0123\right]}$	0	0.0210	*C*	−1	0.0300
$p_{\left[3102\right]}$	2	0.0206	*H*	2	0.0277
$p_{\left[0213\right]}$	2	0.0204	*C*	2	0.0214
$p_{\left[0213\right]}$	3	0.0124	*C*	7	0.0208

## Data Availability

The data supporting the reported results were obtained from publicly available datasets. The EEG data analyzed in this study are available at PhysioNet (https://physionet.org) under the Sleep-EDF Database Expanded.

## Appendix A

### Appendix A.1. Confusion Matrix for Random Forest and Support Vector Machines

This appendix presents the confusion matrices corresponding to the classification algorithms random forest (RF) and support vector machine (SVM). The matrices are calculated using four different feature sets, allowing for the evaluation of each algorithm’s performance in classifying sleep stages.

### Appendix A.2. Performance of Random Forest and Support Vector Machine

Here, we present a table summarizing the performance of the random forest and support vector machine algorithms. The table includes the mean accuracy and standard deviation (SD) for the different feature sets, highlighting their effectiveness in classifying sleep stages.

**Table A1:** Mean accuracy (Acc.) and standard deviation (SD) for the classifiers: random forest (RF) and support vector machine (SVM).

		RF		SVM	
		**Acc**	**SD**	**Acc**	**SD**
Features	PE and C	0.71	0.01	0.72	0.01
	GWPE and GWPEC	0.76	0.01	0.77	0.01
	PDF	0.74	0.01	0.74	0.01
	GWPDF	0.79	0.01	0.78	0.01
